# Dissecção aórtica de tipo B de Stanford: relato de caso e revisão de literatura

**DOI:** 10.1590/1677-5449.000117

**Published:** 2017

**Authors:** Wilson Michaelis, Antônio Lacerda Santos, Rogério Akira Yokohama, Marianne Ariely Andretta, Mariana Vieira Delazari, Luciano Vieira, Erick Fernando Seguro, Lucas Mansano Sarquis

**Affiliations:** 1 Hospital Universitário Evangélico de Curitiba, Cirurgia Vascular, Curitiba, PR, Brasil.

**Keywords:** dissecção aórtica, endovascular, dissecção aórtica de tipo B de Stanford

## Abstract

O complexo tratamento de dissecção da aorta ainda apresenta controvérsias devido à gravidade do caso e à necessidade de individualização da terapêutica. A gravidade relaciona-se ao difícil diagnóstico pelas queixas inespecíficas e pelas graves complicações inerentes à evolução da doença (ruptura aórtica, síndrome de má perfusão, dissecção retrógrada, dor ou hipertensão refratária). Este relato apresenta um homem de 61 anos, tabagista e hipertenso mal controlado, que evoluiu para dissecção aórtica de tipo B de Stanford. Foi abordado através de técnica endovascular com uso de endoprótese com stent para tratamento do caso após falha do tratamento medicamentoso. O tratamento endovascular mostrou-se uma ferramenta eficaz para o tratamento definitivo, com boa taxa de sobrevida ao final do primeiro ano após o procedimento.

## INTRODUÇÃO

A primeira causa de óbito na população mundial tem origem circulatória, incluindo infarto agudo do miocárdio, acidente vascular cerebral, síndrome aórtica aguda, entre outros^1^. A incidência de dissecção aórtica aguda é entre 3-5 casos para cada cem mil habitantes na população dos Estados Unidos da América^2^. As principais causas de dissecção da aorta são hipertensão de longa data, doenças do tecido conjuntivo e trauma^3^.

Os casos de dissecção aórtica de tipo B de Stanford são tratados prioritariamente com suporte clínico, com indicação de cirurgia endovascular ou convencional para os pacientes que apresentarem complicações^4^. Entre as principais complicações, encontram-se ruptura aórtica, síndrome de má perfusão e dissecção retrógrada^5^.

Para os casos de dissecção aórtica de tipo B complicada, mesmo com o tratamento cirúrgico as taxas de óbito são altas, podendo chegar a 29%^6^. A técnica endovascular demanda maior habilidade, porém tem morbimortalidade menor quando comparada ao tratamento cirúrgico convencional. Entretanto, a passagem de fluxo sanguíneo pelo falso lúmen da dissecção pode evoluir para aneurisma e ruptura eventual da aorta^7^.

## RELATO DO CASO

Paciente masculino, 61 anos, foi admitido no pronto-socorro do Hospital Universitário Evangélico de Curitiba com quadro de dor torácica de forte intensidade, com 6 horas de evolução, do tipo facada, irradiada para região interescapular, sem fator de alívio. Tinha história prévia de hipertensão arterial sistêmica sem controle adequado, dislipidemia e obesidade; tabagista: 40 maços/ano. Ao exame físico admissional, apresentava-se em estado geral regular, com pressão arterial (PA) 180 × 120 mmHg, frequência cardíaca de 90 bpm, eupneico, ausculta pulmonar sem alterações, abdome globoso sem massa pulsátil, membro inferior direito com todos os pulsos palpáveis, membro inferior esquerdo com pulso femoral diminuído, demais pulsos ausentes, porém com membro compensado e boa perfusão.

**Figura 1:**
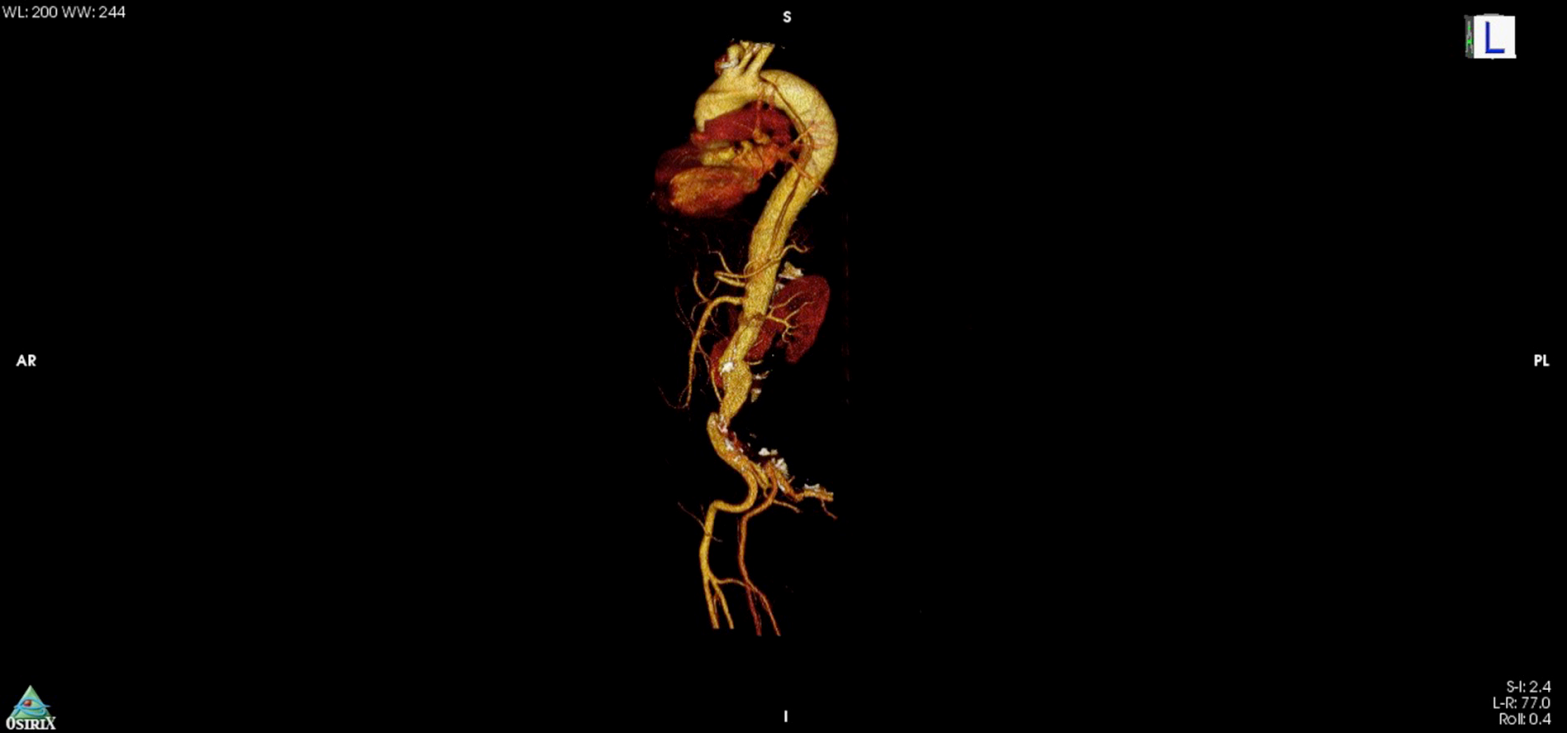
Angiotomografia computadorizada de admissão evidenciando dissecção aórtica de tipo B de Stanford.

Iniciaram-se cuidados intensivos com controle de PA através de nitroglicerina e monitorização em sala de emergência. Após estabilização parcial do quadro clínico, iniciou-se rota para investigação de infarto agudo do miocárdio e optou-se por realizar angiotomografia de tórax, abdome e pelve (Figura 1). O exame evidenciou diagnóstico de dissecção da aorta toracoabdominal, classificada como tipo B de Stanford e tipo III de DeBakey, estendendo-se até as ilíacas comuns com oclusão da artéria ilíaca esquerda.

O paciente foi transferido para a unidade de terapia intensiva (UTI), com alívio na intensidade da dor, mas manutenção do desconforto torácico. Apresentava-se ainda hipertenso, sem possibilidade de retirada de drogas vasoativas.

No sexto dia de internamento, foi realizado tratamento endovascular para corrigir o *flap* proximal, com colocação de endoprótese (Valiant Captiva, Medtronic Vascular®, Santa Rosa, CA, Estados Unidos da América). O procedimento foi realizado sob anestesia geral por acesso femoral sob visão direta após a dissecção da artéria femoral direita. A arteriografia de posicionamento realizada na altura do arco aórtico com cateter *pigtail* confirmou o início da dissecção após a origem da artéria subclávia esquerda, o que permitiu acomodação da prótese sem necessidade de oclusão. Não foi utilizado balão de acomodação devido ao risco de disseção retrógrada. Não houve intercorrências no procedimento, e a arteriografia de controle evidenciou selamento do *flap* proximal.

No pós-operatório (PO) imediato, o doente apresentava pulsos diminuídos do membro inferior esquerdo. Optou-se por aguardar para realização de exame de controle para preservação da função renal, e o paciente foi novamente transferido à UTI.

**Figura 2:**
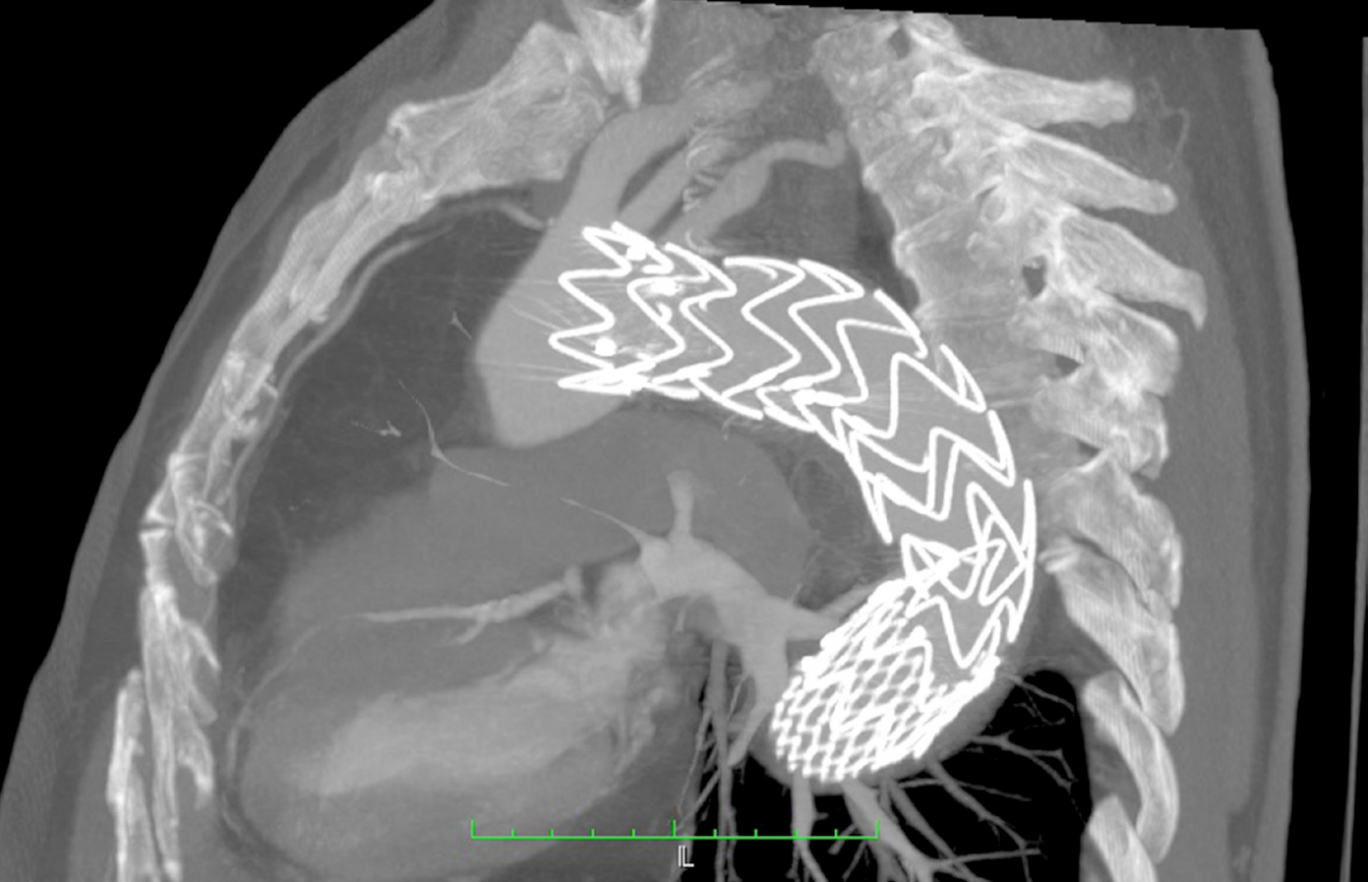
Imagem intraoperatória para correção do *flap* proximal com endoprótese.

No sexto dia após a intervenção, o doente apresentou desconforto abdominal, então optou-se pela realização de nova angiotomografia. Ao exame (Figuras 2 e 3), demonstrou-se selamento do *flap* proximal da dissecção torácica, artérias viscerais emergindo da luz verdadeira e manutenção do *flap* de dissecção infrarrenal, o que preservou a dissecção até as regiões de artérias ilíacas.

**Figura 3:**
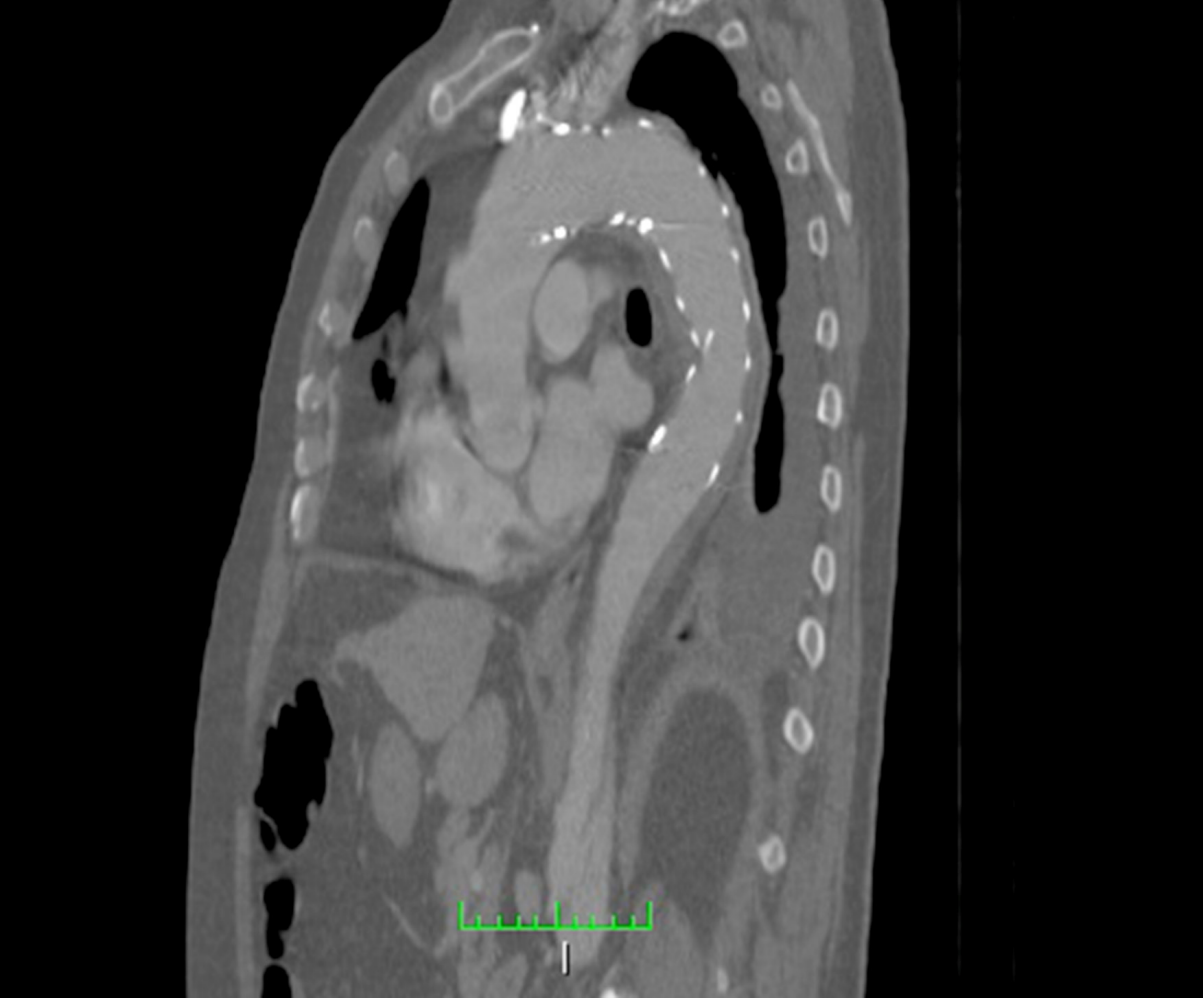
Angiotomografia computadorizada evidenciando selamento do *flap* proximal.

**Figura 4:**
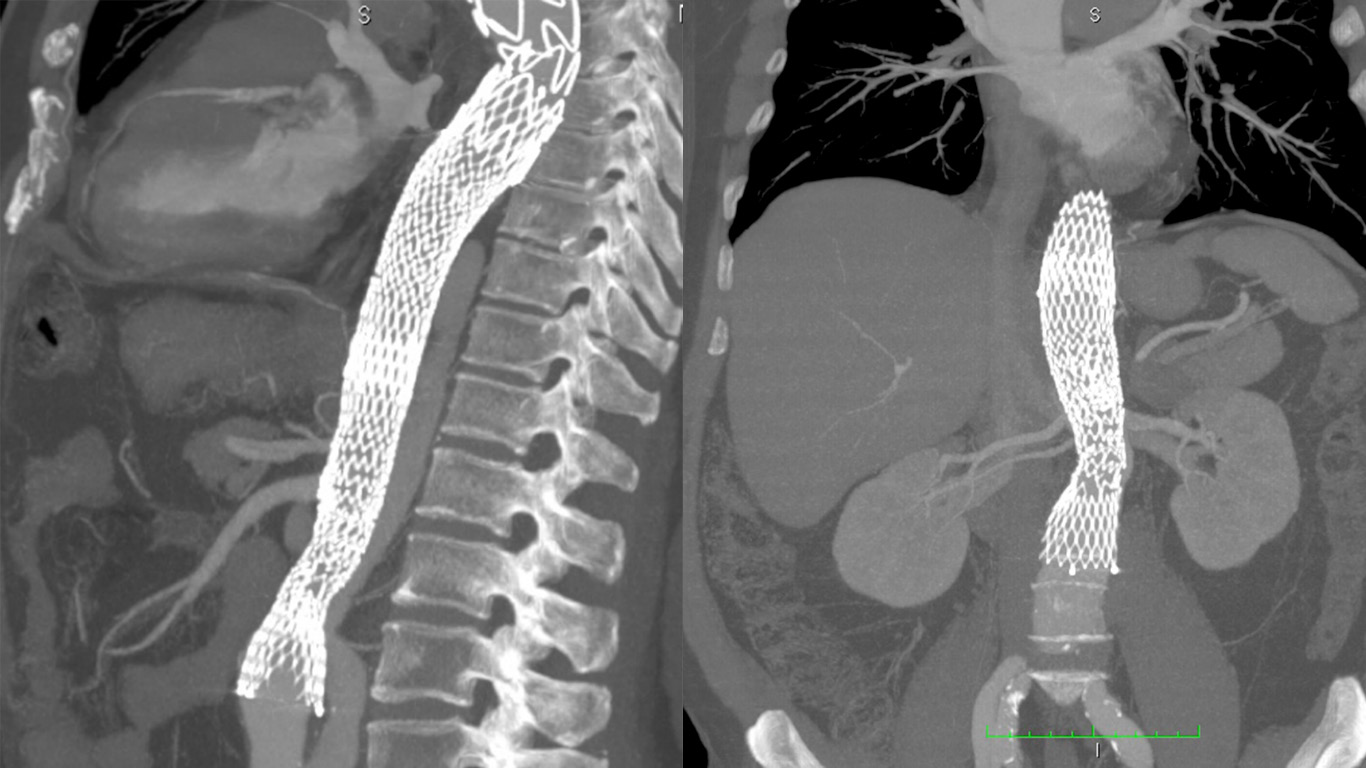
Colocação de endoprótese ao nível das artérias viscerais.

Após a discussão do caso, foi proposta nova abordagem endovascular e colocação de stent não recoberto de aorta (X-EL, JOTEC®, Hechingen, Alemanha) ao nível das artérias viscerais. Os objetivos dessa segunda abordagem foram a expansão da luz verdadeira, a melhora do fluxo das artérias viscerais e a trombose da falsa luz. Além disso, buscou-se reduzir o processo de remodelamento e, consequentemente, a degeneração aneurismática da dissecção aórtica. O procedimento não apresentou nenhuma intercorrência e ocorreu retorno de pulsos no membro inferior esquerdo no PO imediato. A alta do paciente aconteceu no 10º dia de PO após a segunda intervenção. O período de hospitalização, desde o início dos sintomas até a alta, foi de 30 dias. Foi mantido um acompanhamento ambulatorial, sem presença de sintomas nas duas primeiras consultas realizadas, com 15 e 40 dias após a alta. Seguiu em uso de bloqueador de canal de cálcio e betabloqueador. No exame de controle, 40 dias após a última intervenção, não foram evidenciadas complicações do tratamento endovascular (Figuras 4 e 5). Notou-se manutenção da falsa luz por reenchimento distal e dissecção assintomática de artéria ilíaca comum esquerda até a artéria femoral comum esquerda.

**Figura 5:**
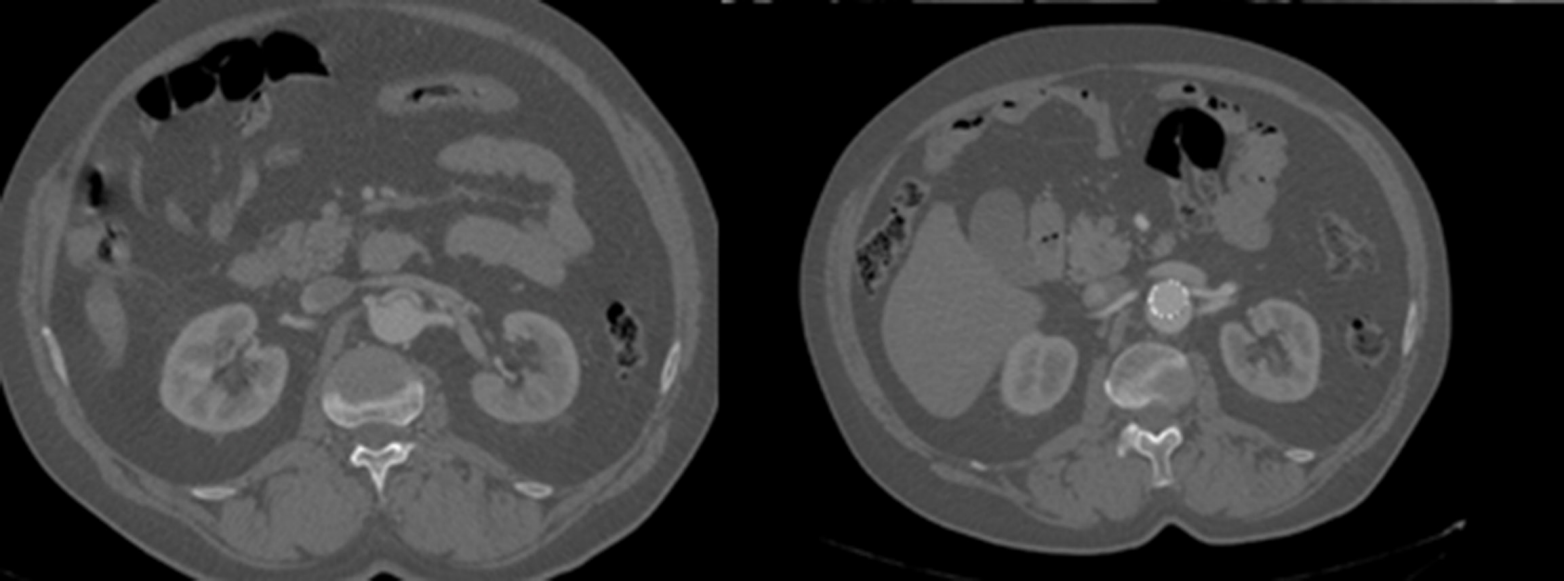
Imagem tomográfica comparativa do pré-operatório e do controle pós-operatório. Manutenção da falsa luz por reenchimento retrógrado e luz verdadeira de maior diâmetro no pós-operatório.

## DISCUSSÃO

A dissecção aórtica é uma doença da camada média do vaso em que o fluxo sanguíneo ocorre entre as camadas média e íntima^8^. A incidência é de três casos para cada 100.000 habitantes por ano, com acometimento por idade bimodal^9^. Há diversos fatores que podem levar à degeneração da camada média e ao surgimento da dissecção, entre eles aterosclerose, hipertensão, tabagismo, sexo masculino e arteriopatias inflamatórias^10^. Assim, o doente do caso relatado apresentava fatores de riscos típicos para o surgimento da dissecção aórtica no segundo pico de incidência: masculino, tabagista e hipertenso.

A dissecção da aorta é avaliada através da classificação de DeBakey ou de Stanford. Stanford classifica de acordo com o acometimento da aorta ascendente (tipo A) ou da aorta descendente (tipo B)^11^. Já a classificação de DeBakey se refere ao acometimento da aorta ascendente, dividida em tipo I (acomete desde a ascendente até a descendente), tipo II (restrita à ascendente) e tipo III (acomete a descendente)^12^.

O diagnóstico de dissecção aórtica é sempre complexo, seja pela baixa incidência ou procura de serviços médicos, seja pela apresentação inespecífica, com primeiro diagnóstico equivocado em 38% dos casos na avaliação inicial^13^. A dor torácica aguda com irradiação para o dorso é o principal sintoma encontrado, associado a quadro hipertensivo, bradicardia e síncope^14^. Sintomas menos comuns são dor abdominal, déficit neurológico, síndrome de Horner e paralisia de cordas vocais^10^. No presente relato, a admissão com suspeita inicial de infarto agudo do miocárdio reforça a complexidade do diagnóstico no início do atendimento, mesmo com a presença dos sintomas mais comuns.

A solicitação de exames complementares é fundamental para definir o diagnóstico e determinar a extensão da dissecção e sinais de gravidade. Os principais exames solicitados, em ordem decrescente, são: angiotomografia computadorizada (ATC), ressonância nuclear magnética (RNM), ecocardiograma transesofágico (ETE) e arteriografia^15^.

A ATC com contraste é uma opção viável e rápida para a maioria dos centros de emergência, além de ter sensibilidade de até 95% e especificidade entre 85-95%^10^. Já a RNM apresenta aproximadamente 100% de sensibilidade e especificidade, além de não necessitar de contraste intravenoso nem expor à radiação^16^. O uso de ETE está relacionado principalmente a casos de dissecção de tipo A, além de ter capacidade de identificar acometimento de artérias coronárias, tamponamento cardíaco e insuficiência aórtica^17^. O paciente em questão foi submetido a ATC como exame de escolha, o que possibilitou firmar o diagnóstico e realizar o planejamento terapêutico.

A terapêutica inicial é fundamental para diminuir a agressão à parede da aorta, através da diminuição da PA e controle da frequência cardíaca. Em casos em que a PA sistólica se mantém acima de 100-120 mmHg após o uso de betabloqueadores, há indicação do uso de medicamentos vasodilatadores, como nitroprussiato de sódio ou nitroglicerina^8^. Em associação ao vasodilatador, deve-se utilizar analgésicos, na maioria das vezes opioides, para alívio sintomático e adjuvante no controle da PA e frequência cardíaca^10^. Devido à emergência hipertensiva em que o paciente estava ao ser admitido no hospital, a escolha pela nitroglicerina se mostrou eficaz e condizente com a literatura^8^.

As condutas cirúrgicas na fase aguda da dissecção são reservadas para casos em que haja complicações. É preferível realizá-las após estabilização do quadro, devido à mortalidade de até 34% ao se indicar tratamento cirúrgico na fase aguda^15^^,^ 18. A técnica endovascular é preferível à técnica aberta pela menor morbidade^19^, e pode-se optar por fenestrações endovasculares com o objetivo de comunicar a falsa com a verdadeira luz e despressurizar a primeira. Já a associação de stent promove estabilidade da artéria e passagem de sangue pelo lúmen verdadeiro, diminuindo consideravelmente as complicações da dissecção^20^. Nos casos de isquemia, o uso de stent aórtico não recoberto com técnica de Petticoat, embora não esteja bem estabelecido, vem mostrando benefícios na literatura.

Estudos demonstram que as principais complicações associadas ao tratamento endovascular com endoprótese e stent são persistência do falso lúmen, ruptura aórtica, acidente vascular encefálico (AVE), paraplegia e dissecção retrógrada^20^^,^ 21. Estudos multicêntricos indicam que a taxa de sucesso pode ser de até 89% com apenas um procedimento, com taxas de complicações variáveis entre 180 pacientes – AVE: 3,9% (sete pacientes); e paraplegia: 2,8% (cinco pacientes)^22^. Quanto à taxa de sobrevida, indica-se que o período crítico para o óbito está nos primeiros 30 dias após o procedimento, com mortalidade entre 5-16%. Há uma sobrevida maior ao final do primeiro ano, variando entre 89-95%^23^^,^ 24.

O tratamento com stent tem o papel de evitar a má perfusão e diminuir a taxa de degeneração aneurismática da falsa luz. No presente caso, embora mantida a falsa luz por reenchimento distal, notou-se sua diminuição nos exames de controle^8^. O uso de betabloqueador e bloqueadores dos canais de cálcio é relatado como fator protetor da ruptura e crescimento da falsa luz. O risco de rotura da falsa luz está relacionado à idade, ao *flap* de disseção na concavidade do arco aórtico e ao diâmetro da falsa luz^25^.
