# Supplementary material for: Dissecção aórtica de tipo B de Stanford: relato de caso e revisão de literatura
Source: J Vasc Bras. 2017 Jul-Sep;16(3):252–7. [Article in Portuguese] doi: 10.1590/1677-5449.000117 (PMC5868944; doi:10.1590/1677-5449.000117)
Supplement: Translation

# Translation: Stanford B aortic dissection: case report and literature review

**Authors:** Wilson Michaelis, Antônio Lacerda Santos, Rogério Akira Yokohama, Marianne Ariely Andretta, Mariana Vieira Delazari, Luciano Vieira, Erick Fernando Seguro, Lucas Mansano Sarquis

**Affiliations:** 1 Hospital Universitário Evangélico de Curitiba, Cirurgia Vascular, Curitiba, PR, Brazil.

**Keywords:** aortic dissection, endovascular, Stanford type B aortic dissection

## Abstract

Complex treatment of aortic dissection is still a controversial subject because of the severity of these cases and the need to treat on a case-by-case basis. Severity is related to the difficulty of diagnosis caused by nonspecific complaints and by the serious complications inherent to disease progression (aortic rupture, hypoperfusion syndrome, retrograde dissection, refractory hypertension or pain). This article reports the case of a 61-year-old male smoker with poorly controlled hypertension who suffered a Stanford type B aortic dissection. After drug-based treatment failed, the patient was treated using endovascular techniques to place an endoprosthesis with stenting. Endovascular treatment is proving to be an effective tool for definitive treatment, with a good survival rate at the end of the first year after the procedure.

## INTRODUCTION

The number one cause of death in the global population is of circulatory origin, including acute myocardial infarction, stroke, acute aortic syndrome, and others.^1^ The incidence of acute aortic dissection among the population of the United States is 3-5 cases in every hundred thousand inhabitants.^2^ The most common causes of dissection of the aorta are long term hypertension, connective tissue diseases, and trauma.^3^

Cases of aortic dissection classified as Stanford type B are preferably treated with clinical support and endovascular or conventional surgery are reserved for patients who exhibit complications.^4^ The most important complications include aortic rupture, hypoperfusion syndrome, and retrograde dissection.^5^

Even with surgical treatment, type B aortic dissection with complications is associated with elevated mortality rates, which can be as high as 29%.^6^ The endovascular technique requires greater skill, but it is associated with lower morbidity and mortality than conventional surgical treatment. However, blood flow through the false lumen created by dissection can progress to aneurysm formation and sometimes to rupture of the aorta.^7^

## CASE REPORT

A 61-year-old male patient was admitted via the emergency department to the Hospital Universitário Evangélico de Curitiba (Brazil) with strong pain in the chest with onset 6 hours previously. He described the pain as “stabbing”, radiating to the interscapular area, and with no alleviating factors. He had a prior history of poorly controlled systemic arterial hypertension, dyslipidemia, obesity, and a 40 pack-years smoking habit. Physical examination on admission, revealed regular general condition, arterial blood pressure (BP) of 180 × 120 mmHg, heart rate of 90 bpm, eupnea, unremarkable pulmonary auscultation, distended abdomen free from pulsating masses, right lower limb with all pulses palpable, left lower limb with weak femoral pulse and all other pulses absent, but the limb was not decompensated and had good perfusion.

Critical care was initiated, controlling BP with nitroglycerine and monitoring the patient in an emergency room. After partial stabilization of clinical status, work-up for investigation of acute myocardial infarction was initiated with angiotomography of the chest, abdomen, and pelvis (Figure 1). This examination revealed evidence for a diagnosis of dissection of the thoracoabdominal aorta, classified as Stanford type B and DeBakey type III, extending as far as the common iliacs, and with occlusion of the left iliac artery.

**Figure 1:** Computed tomography angiography at admission showing Stanford type B aortic dissection.

The patient was transferred to an intensive care unit (ICU), with alleviation of the intensity of pain, but with persistent thoracic discomfort. He was still hypertensive, with no possibility of withdrawing the vasoactive drugs.

On the sixth day in hospital, endovascular techniques were used to repair the proximal flap, with placement of a stent graft (Valiant Captiva, Medtronic Vascular®, Santa Rosa, CA, USA). This procedure was conducted under general anesthesia via a femoral access and with direct observation, after dissection of the right femoral artery. Positional arteriography at the height of the aortic arch with a pigtail catheter confirmed that the dissection began after the origin of the left subclavian artery, enabling placement of the stent without occlusion. A balloon was not used for stent fitting because of the risk of retrograde dissection. There were no intercurrent conditions during the procedure and control arteriography showed that the proximal flap had been sealed.

During the immediate postoperative period, the patient had weakened pulses in the left lower limb. The decision was therefore taken to delay the control examination to preserve renal function, and the patient was once more transferred to the ICU.

On the sixth day after the intervention, the patient complained of abdominal discomfort and so angiotomography was conducted once more. The examination (Figures 2 and 3) showed that the proximal flap of the thoracic dissection had been sealed, with the visceral arteries emerging from the true lumen, and the infrarenal dissection flap remained, maintaining the dissection as far as the iliac arteries.

**Figure 2:** Intraoperative image during repair of the proximal flap with a stent graft.

**Figure 3:** Computed tomography angiography showing that the proximal flap has been sealed.

After discussion of the case, it was proposed that another endovascular approach would be made to place an uncovered aorta stent (X-EL, JOTEC®, Hechingen, Germany) at the level of the visceral arteries. The objectives of this second operation were to expand the true lumen, improve flow through the visceral arteries and provoke thrombosis of the false lumen. It was also intended to arrest the process of remodeling and, consequently, aneurysmal degeneration of the aortic dissection. This procedure was completed with no intercurrent events and left lower limb pulses returned in the immediate postoperative period. The patient was discharged 10 days after the second intervention. The entire hospital stay, from onset of symptoms to discharge, was 30 days. He was assigned to outpatients follow-up, with no symptoms detected at the first two consultations, at 15 and 40 days after discharge. He was maintained on calcium channel blockers and beta blockers. A control examination 40 days after the last intervention did not reveal any complications from endovascular treatment (Figures 4 and 5). It was noted that the false lumen remained, with distal refilling and asymptomatic dissection of the left common iliac artery to the left common femoral artery.

**Figure 4:** Placement of a stent at the level of the visceral arteries.

**Figure 5:** Comparison of preoperative and postoperative control tomographic images. In the postoperative image, the false lumen is maintained by retrograde refilling and the diameter of the true lumen has increased.

## DISCUSSION

Aortic dissection is a disease of the tunica media of the vessel allowing blood to flow between the media and intima layers.^8^ Incidence is three cases for every 100,000 inhabitants per year, with a bimodal age distribution.^9^ There are many factors that can lead to degeneration of the tunica media and to occurrence of dissection, including atherosclerosis, hypertension, smoking, male sex, and inflammatory arteriopathies.^10^ The patient in the case described here therefore had the typical risk factors for the second peak of aortic dissection incidence: male sex, smoking, and hypertension.

Dissections of the aorta are evaluated according to the DeBakey or Stanford classifications. The Stanford classification is according to involvement of the ascending aorta (type A) or the descending aorta (type B).^11^ The DeBakey classification refers to involvement of the ascending aorta, divided into type I (both descending and ascending involved), type II (restricted to the ascending), or type III (involving the descending aorta).^12^

Diagnosis of aortic dissection is always complex, whether because of its low incidence or because few people seek medical services, or whether because of the nonspecific presentation, with erroneous first diagnosis at initial assessment in 38% of cases.^13^ Acute chest pain radiating to the back is the principal symptom observed, combined with hypertension, bradycardia, and syncope.^14^ Less common symptoms include abdominal pains, neurological deficits, Horner syndrome, and paralysis of the vocal cords.^10^ In the case described here, admission with initial suspicion of acute myocardial infarction underscores the complexity of diagnosis at initial examination, even when the most common symptoms are present.

Supplementary examinations are extremely important for definition of diagnosis and to determine the extent of dissection and signs of severity. The most important examinations, in descending order, are: computed tomography angiography (CTA), magnetic resonance imaging (MRI), transesophageal echocardiogram (TEE), and arteriography.^15^

Computed tomography angiography with contrast is a rapid method that is viable in the majority of emergency centers, offering sensitivity of up to 95% and specificity of 85-95%.^10^ In turn, MRI offers approximately 100% sensitivity and specificity and does not need intravenous contrast or exposure to radiation,^16^ while TEE is primarily used in relation to type A dissection cases, with the capacity to identify involvement of coronary arteries, cardiac tamponade, and aortic insufficiency.^17^ The patient in question was examined with CTA as the first choice option, resulting in confirmation of the diagnosis and enabling treatment planning.

Initial treatment is directed at attenuating the aggression suffered by the aorta wall, by reducing BP and controlling heart rate. Vasodilators such as sodium nitroprusside or nitroglycerine are indicated in cases in which systolic BP still exceeds 100-120 mmHg after administration of beta blockers.^8^ In combination with a vasodilator, analgesics should be given (opioids in the majority of cases) to relieve symptoms and aid in control of BP and heart rate.^10^ Given the hypertensive emergency condition in which the patient was admitted to hospital, the choice of nitroglycerine proved effective and is in line with the literature.^8^

During the acute phase of dissection, surgery is reserved for cases in which there are complications. It is preferable to delay surgery until the patient has been stabilized, since mortality is as high as 34% when surgical treatment is initiated during the acute phase.^15^^,^ 18 Endovascular techniques are preferred to open methods because morbidity is lower^19^, and endovascular fenestrations can be used to provide communication between the false and true lumens, to depressurize the false lumen. Placement of a stent increases stability of the artery and facilitates blood flow through the true lumen, considerably reducing the complications caused by dissection.^20^ Although use of an uncovered aortic stent with the Petticoat technique is not yet well-established, there are reports in the literature showing it can be beneficial in cases with ischemia.

Studies demonstrate that the most important complications associated with endovascular treatment with endoprostheses and stents are persistence of a false lumen, aortic rupture, strokes, paraplegia, and retrograde dissection.^20^^,^ 21 A multicenter study showed that the success rate can be as high as 89% with just a single procedure, with variable complications across 180 patients – stroke: 3.9% (seven patients); and paraplegia: 2.8% (five patients).^22^ With regard to survival rate, it appears that the critical period for mortality is the first 30 days after the procedure, with rates of 5 to 16%. There is greater survival at the end of the first year, varying from 89 to 95%.^23^^,^ 24

The role of treatment with stents is to avoid hypoperfusion and reduce the rate of aneurysmal degeneration of the false lumen. In the case described here, although the false lumen was maintained by distal refilling, control examinations showed that it was reducing.^8^ Beta blockers and calcium channel blockers are described as factors of protection against rupture and expansion of the false lumen. The risk of false lumen rupture is related to age, to dissection *flaps* within the concave portion of the aortic arch, and to the diameter of the false lumen.^25^
